# Supplementary material for: Antioxidant and Anticancer Potential of the New Cu(II) Complexes Bearing Imine-Phenolate Ligands with Pendant Amine N-Donor Groups
Source: Pharmaceutics. 2023 Jan 22;15(2):376. doi: 10.3390/pharmaceutics15020376 (PMC9960331; doi:10.3390/pharmaceutics15020376)
Supplement: Supplementary file 1 [file pharmaceutics-15-00376-s001.zip › pharmaceutics-2147328-supplementary.pdf]

Supporting Information

Antioxidant and Anticancer Potential of the New Cu(II) Complexes Bearing Imine-Phenolate Ligands with Pendant Amine N-Donor Groups

Adriana Castro Pinheiro [1,2], Ianka Jacondino Nunes [2], Wesley Vieira Ferreira [2], Paula Pellenz Tomasini [1], Cristiano Trindade [1,3], Carolina Cristóvão Martins [4], Ethel Antunes Wilhelm [4], Robson da Silva Oliboni [2], Paulo Augusto Netz [5], Rafael Stieler [6], Osvaldo de Lazaro Casagrande, Jr. [6] and Jenifer Saffi [1,*]

[1] Laboratory of Genetic Toxicology, Department of Basic Health Sciences, Federal University of Health Sciences of Porto Alegre (UFCSPA), Porto Alegre 90050-170, RS, Brazil
[2] Group of Catalysis of Theoretical Studies, Center of Chemical, Pharmaceutical and Food Science Center, Federal University of Pelotas (UFPel), Pelotas 96160-000, RS, Brazil
[3] Centro de Investigaciones en Ciencias de la Vida, Universidad Simón Bolívar, Barranquilla 080002, Colombia
[4] Laboratory in Biochemical Pharmacology, Center of Chemical, Pharmaceutical and Food Sciences, Federal University of Pelotas (UFPel), Pelotas 96160-000, RS, Brazil
[5] Grupo de Química Teórica, Instituto de Química, Universidade Federal do Rio Grande do Sul (UFRGS), Porto Alegre 91501-970, RS, Brazil
[6] Laboratory of Molecular Catalysis, Instituto de Química, Universidade Federal do Rio Grande do Sul (UFRGS), Porto Alegre 91501-970, RS, Brazil
* Correspondence: jenifers@ufcspa.edu.br

Table of Contents

Figure S1. ^1H NMR spectrum (400 MHz, $CDCl_3$, 298 K) of **1a**

Figure S2. ^{13}C NMR spectrum (100 MHz, $CDCl_3$, 298 K) of **1a**

Figure S3. ^1H NMR spectrum (400 MHz, $CDCl_3$, 298 K) of **1b**

Figure S4. ^{13}C NMR spectrum (100 MHz, $CDCl_3$, 298 K) of **1b**

Figure S5. ESI-HRMS spectrum for complex **2a**.

Figure S6. ESI-HRMS spectrum for complex **2b**.

Figure S7. ESI-HRMS spectrum for complex **2b** (isotopic profile).

Figure S8. UV-Vis spectra of **1a** and its complex **2a** (1×10^{-4} M) in dichoromethane.

Figure S9. UV-Vis spectra of **1b** and its complex **2b** (1×10^{-4} M) in dichoromethane.

Figure S10. Intermolecular hydrogen bonds between two mononuclear species of complex 2a (thermal ellipsoids drawn at 60% probability level). For clarity the hydrogen atoms not-involved in the hydrogen bonds have been omitted. (N(3)-H(3A)···Cl(2)#2, d(H···A) = 2.24 Å, d(D···A) = 3.2036(17) Å, <(D-H···A) = 160.2 °) (#1 = 2-x, 1-y, 1-z; #2 = 1-x, -y, 1-z).

Figure S11. UV-Vis spectra of **1a** (1 × 10-7 M) in DMSO (250 – 800 nm).

Figure S12. UV-Vis spectra of **1b** (1×10^{-7} M) in DMSO (250 – 800 nm).

Figure S13. UV-Vis spectra of **2a** (1×10^{-7} M) in DMSO (250 – 1000 nm).

Figure S14. UV-Vis spectra of **2b** (1×10^{-7} M) in DMSO (250 – 1000 nm).

Figure S15. UV-Vis spectra of **2a** (1×10^{-7} M) in H_2O (250 – 1000 nm).

Figure S16. UV-Vis spectra of **2b** (1×10^{-7} M) in H_2O (250 – 1000 nm).

Figure S17. Cyclic voltammograms of ligands **1a**-**b** and Cu(II) complexes **2a**-**b**, in DMF solution at 100 mV/s scan rate, corresponding to the **1a** (a), **2a** (b), **1b** (c) and **2b** (d).

Figure S18. Effect of ligands and Cu(II) complexes represented by the **1a** (a), **2a** (b), **1b** (c), **2b** in different concentrations on DPPH radical-scavenger activity. Data are expressed as the mean ± SEM for three independent experiments, carried out on different days. The results are calculated as % of control of the absorbance at 517 nm. Asterisk denotes significance levels when compared to control group: (*) $P < 0.05$, (***) $P < 0.001$ and (****) $P < 0.0001$ (One-way ANOVA followed by the Newman-Keul's test).

Figure S19. Effect of ligands and Cu(II) complexes represented by the **1a** (a), **2a** (b), **1b** (c), **2b** in different concentrations on $ABTS^{+}$ radical-scavenger activity. Data are expressed as the mean ± SEM for three independent experiments, carried out on different days. The results are calculated as % of control of the absorbance at 730 nm. Asterisk denotes significance levels when compared to control group: (*) $P < 0.05$, (***) $P < 0.001$ and (****) $P < 0.0001$ (One-way ANOVA followed by the Newman-Keul's test).

Figure S20. Comparison of the dose-response survival diagrams of MRC-5, MCF-7 and

SW620 cell lines exposed to **1a** and **1b** free ligand (0 – 100 μM) for 24 h or 72 h. The obtained values represent an average of at least three independent experiments. NC represents the negative control. Graphs represent the average ± SD. Statistical analysis were performed using One-way ANOVA followed by Dunnett's multiple comparison test and $p < 0.05$ was considered as significative. * p = 0.0189 (d), 0.0145 (e), 0.0224 (f); ** p = 0.0027 (a), 0.0015 (d); *** p = 0.0010 (c) and (f), 0.0003 (d); **** p = <0.0001.

Figure S21. Comparison of the dose-response survival diagrams of MRC-5, MCF-7 and SW620 cell lines exposed to complex **2a** (0 – 100 μM) for 24 h and 72 h. The obtained values represent an average of at least three independent experiments. NC represents the negative control. Graphs represent the average ± SD. Statistical analysis were performed using One-way ANOVA followed by Dunnett's multiple comparison test and $p < 0.05$ was considered as significative. * p = 0.0141 (a); 0.0185 (24 h - b), 0.0445 (72 h –b), ** p = 0.0026 (b), 0.0036 (24 h - c), 0.0017 (72 h – c) *** p = 0.0002 (a); 0.0009 (24 h-b), 0.0004 (72 h –b), **** p = <0.0001.

Figure S22. Colony Survival for **1a**, **1b**, **2a** and **2b** on MRC-5, MCF-7 and SW620. Data represent mean and SD of 3 independent experiments. One-way ANOVA and Dunnett1s posttest. (*)p<0.05; (**) p<0.01; (***) p<0.001.

Figure S23. Two-dimensional interaction diagram of complexes (a) **2a** and (b) **2b** with DNA (PDB: 1BDNA).

Figure S24. (a) An alternative docking pose for **2b** with an affinity value of –7.4 kcal mol^{-1}. (b) Two-dimensional interaction diagram of this mode with DNA.

Table S1. Crystallographic data and structure refinement parameters for **2a**.

Table S2. Selected geometrical properties of compounds **2a**, **2a′** and **2b** at the B3LYP/def2-TZVPP/def2-SVP level of theory. Distances are in angstroms and angles in degrees.

Coordinates xyz : Coordinates from ORCA-job **2a.**

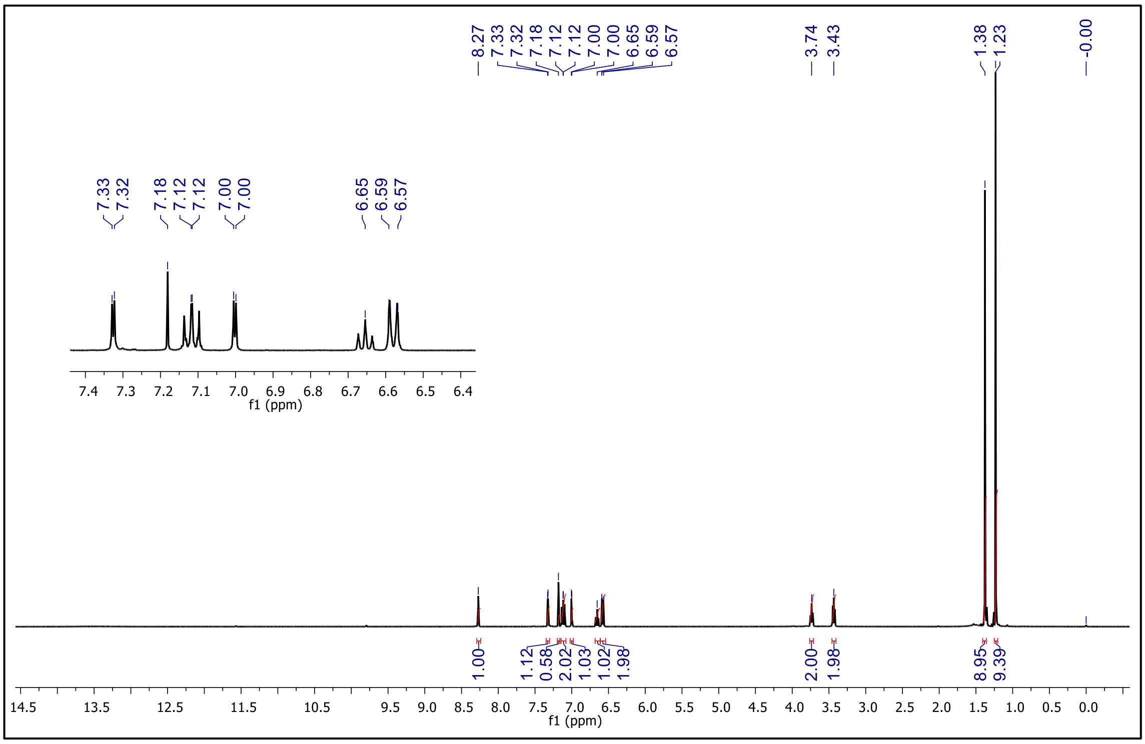

Figure S1. ^{1}HNMR (400 MHz, $CDCl_3$, rt) spectrum of ligand **1a**.

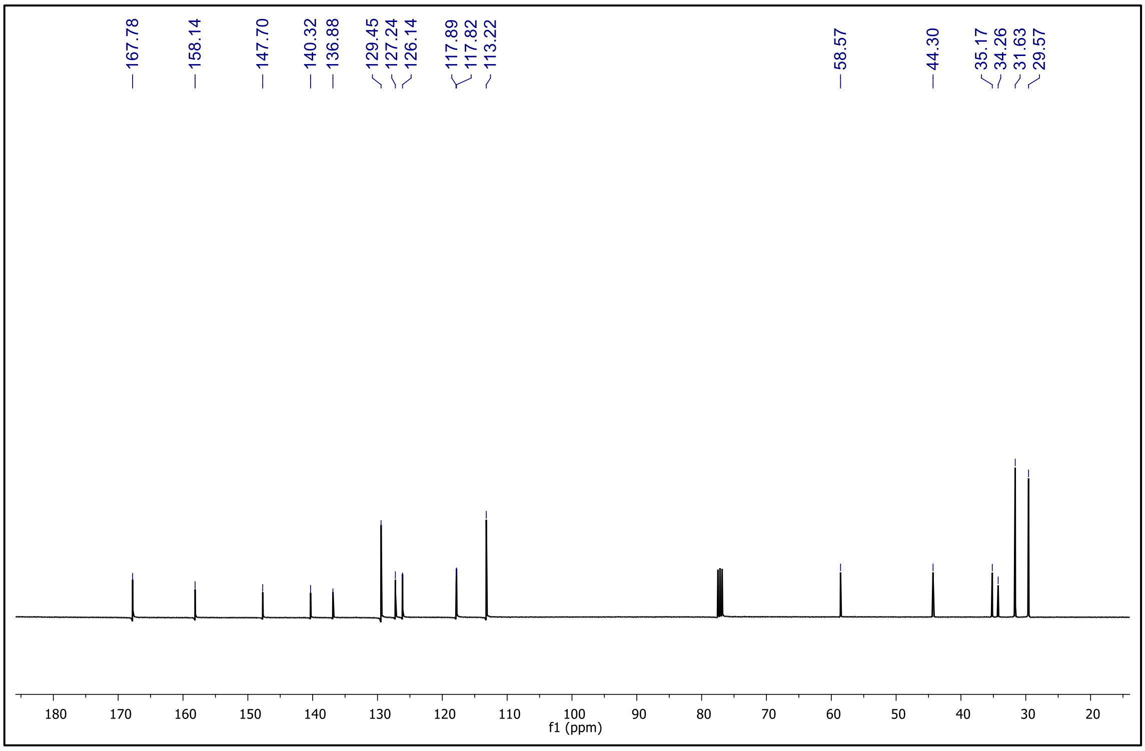

Figure S2. $^{13}C\{^{1}H\}$ NMR (101 MHz, $CDCl_3$, rt) spectra of ligand **1a**.

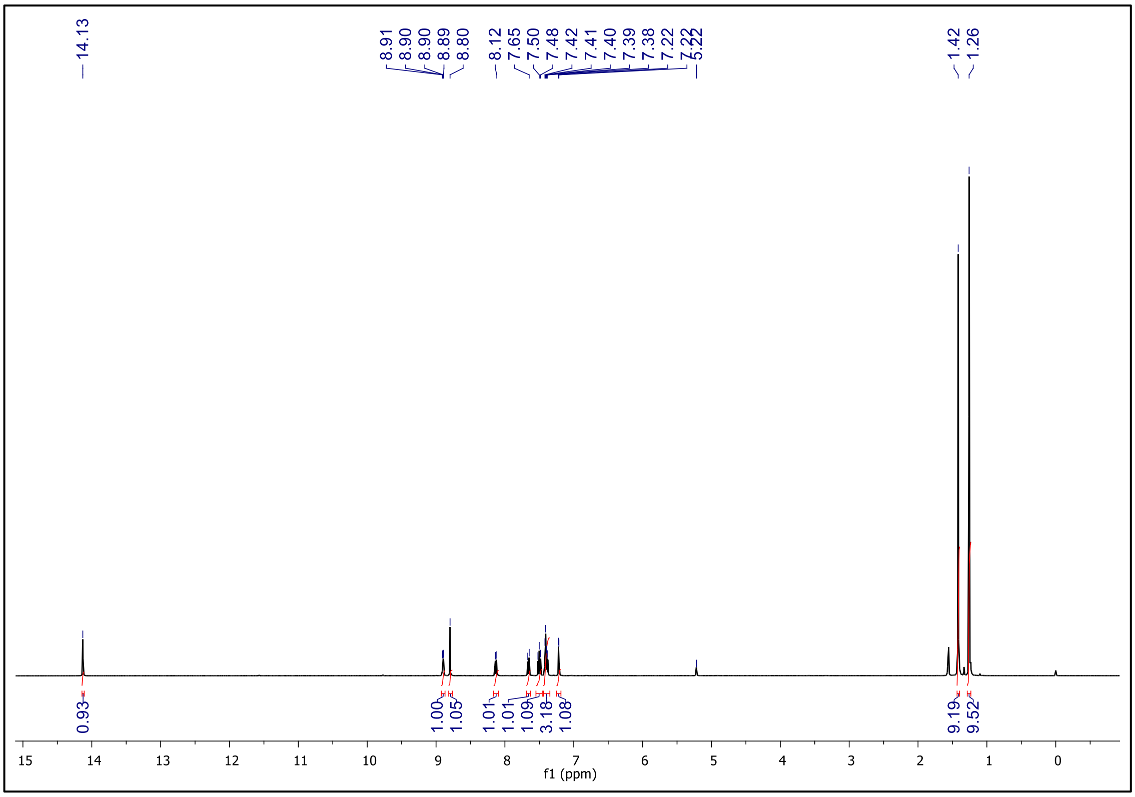

Figure S3. ^{1}HNMR (400 MHz, $CDCl_3$, rt) spectrum of ligand **1b**.

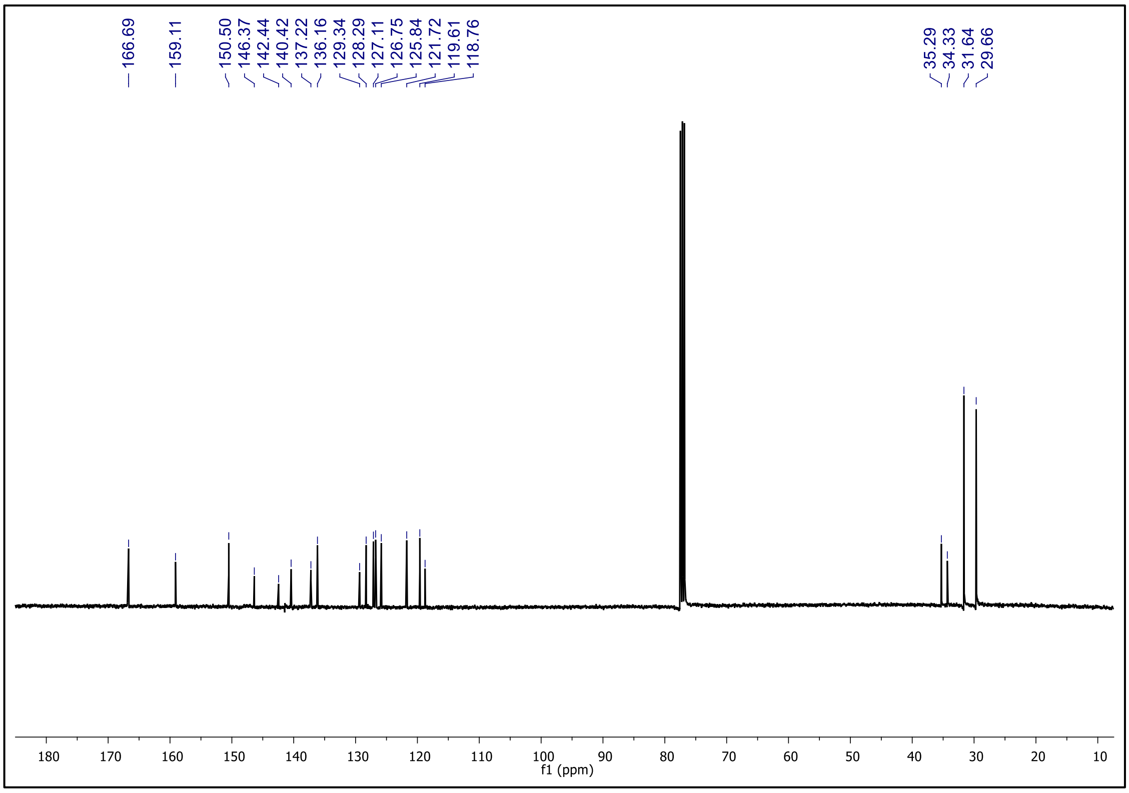

Figure S4. $^{13}C\{^{1}H\}$ NMR (101 MHz, $CDCl_3$, rt) spectra of ligand **1b**.

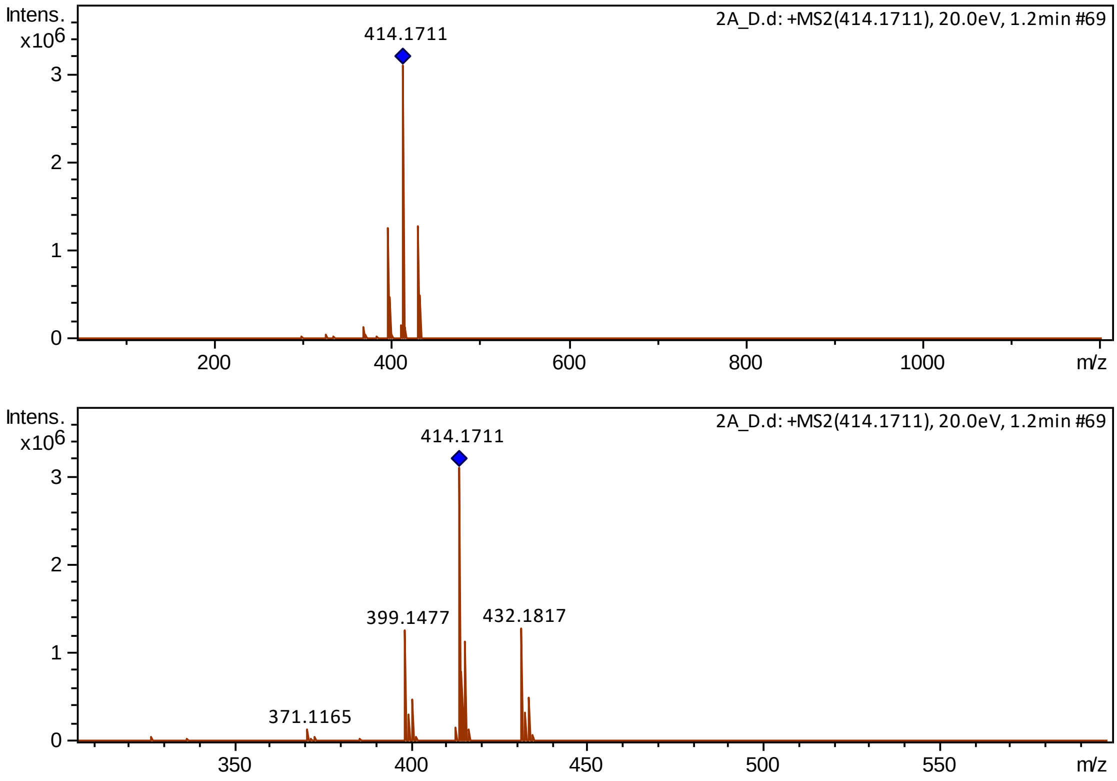

Figure S5. ESI-HRMS spectrum for complex **2a**.

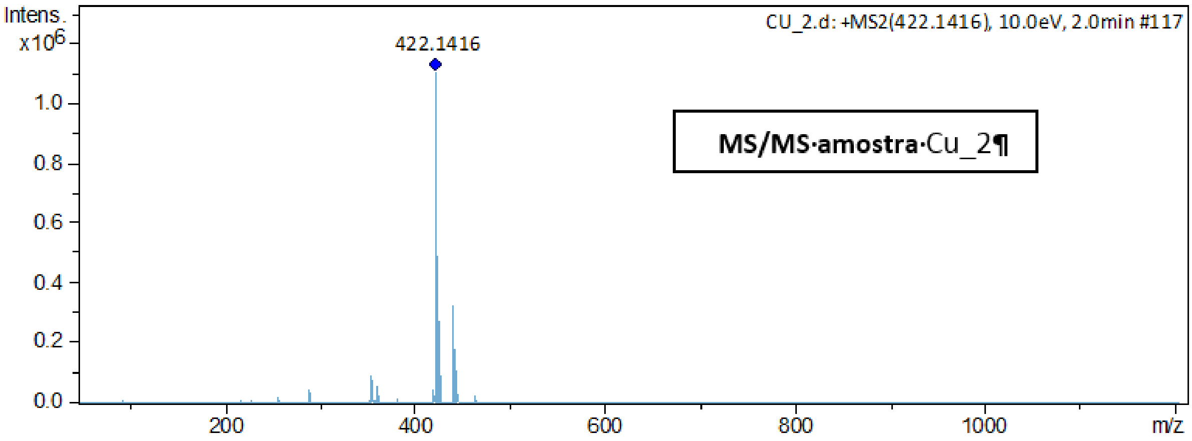

Figure S6. ESI-HRMS spectrum for complex **2b**.

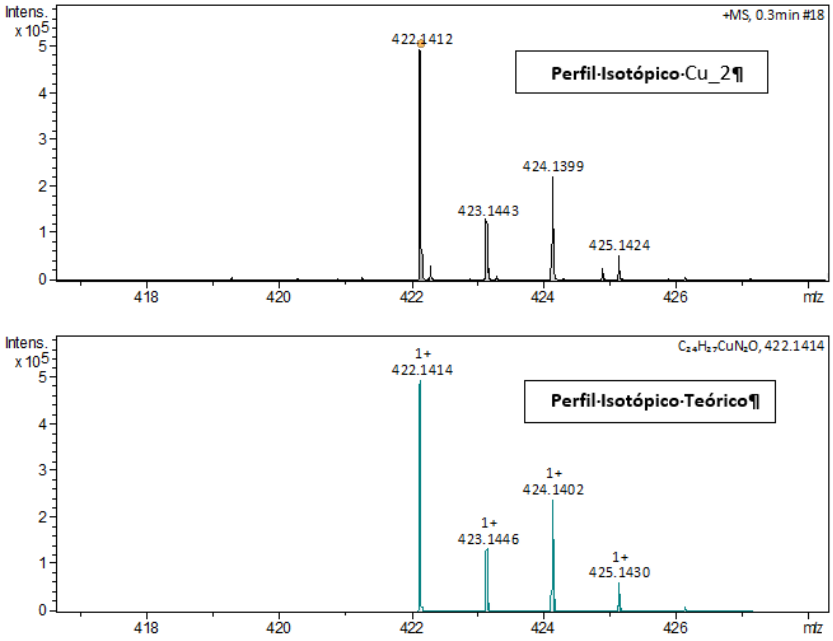

Figure S7. ESI-HRMS spectrum for complex **2b** (isotopic profile).

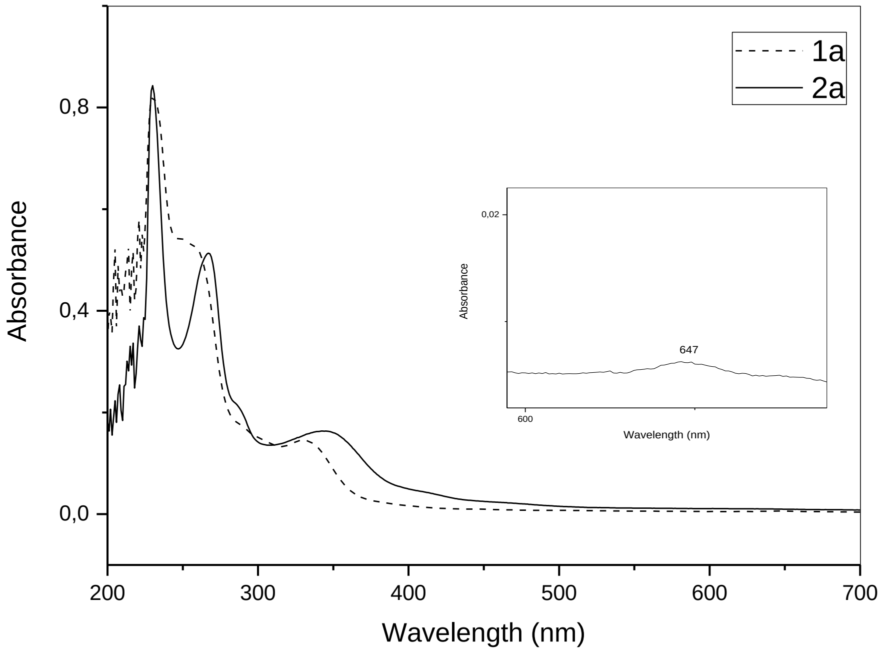

Figure S8. UV-Vis spectra of **1a** and its complex **2a** (1×10^{-4} M) in dichoromethane.

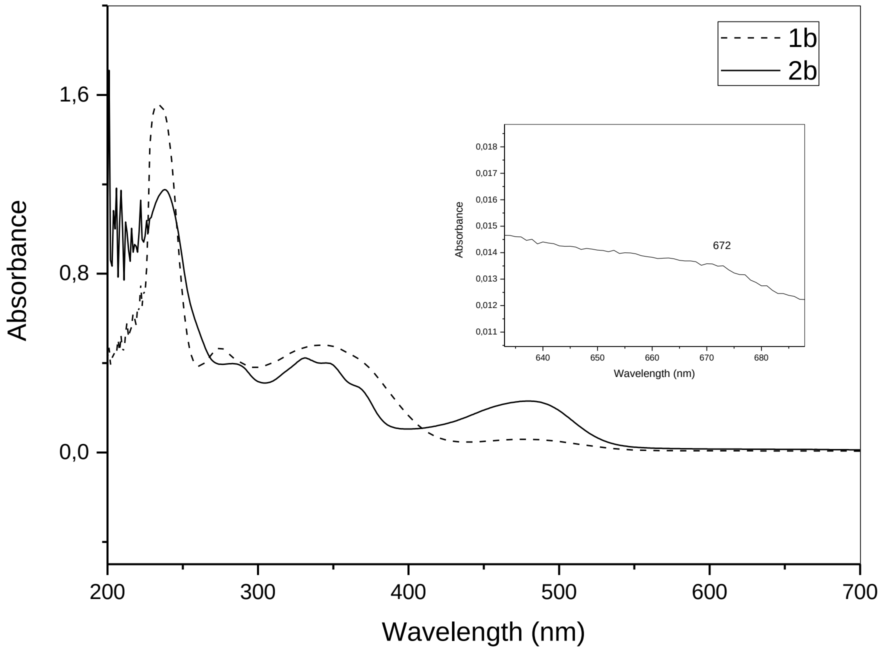

Figure S9. UV-Vis spectra of **1b** and its complex **2b** (1×10^{-4} M) in dichoromethane.

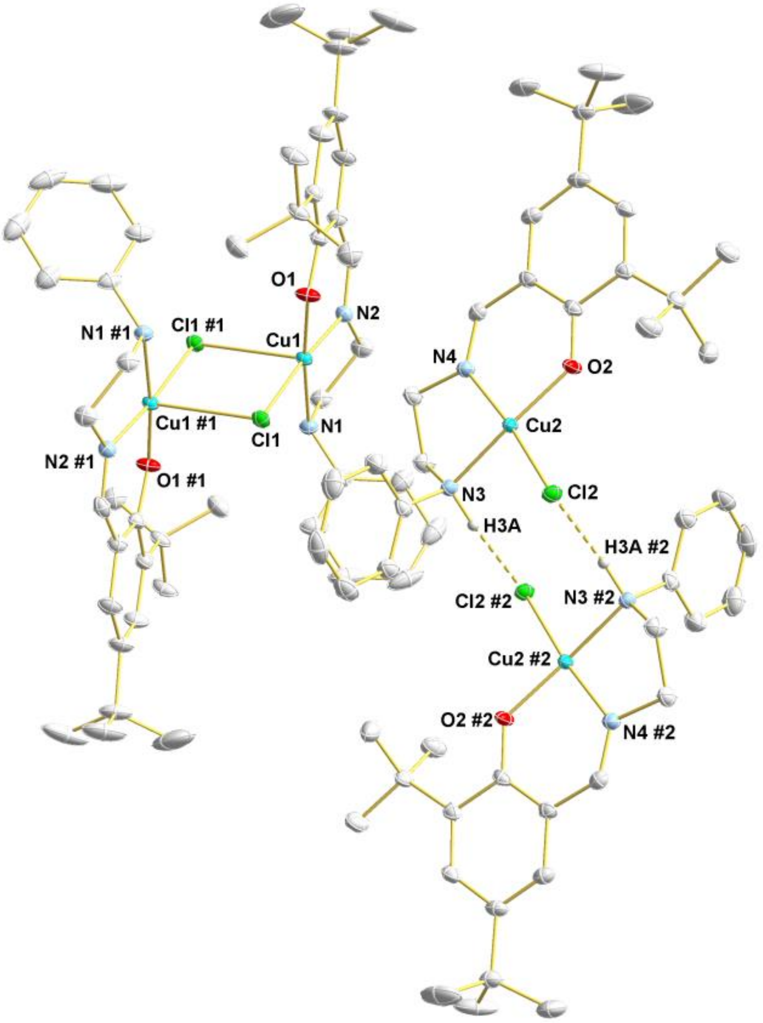

Figure S10. Intermolecular hydrogen bonds between two mononuclear species of complex 2a (thermal ellipsoids drawn at 60% probability level). For clarity the hydrogen atoms not-involved in the hydrogen bonds have been omitted. (N(3)-H(3A)···Cl(2)#2, d(H···A) = 2.24 Å, d(D···A) = 3.2036(17) Å, <(D-H···A) = 160.2 °) (#1 = 2-x, 1-y, 1-z; #2 = 1-x, -y, 1-z).

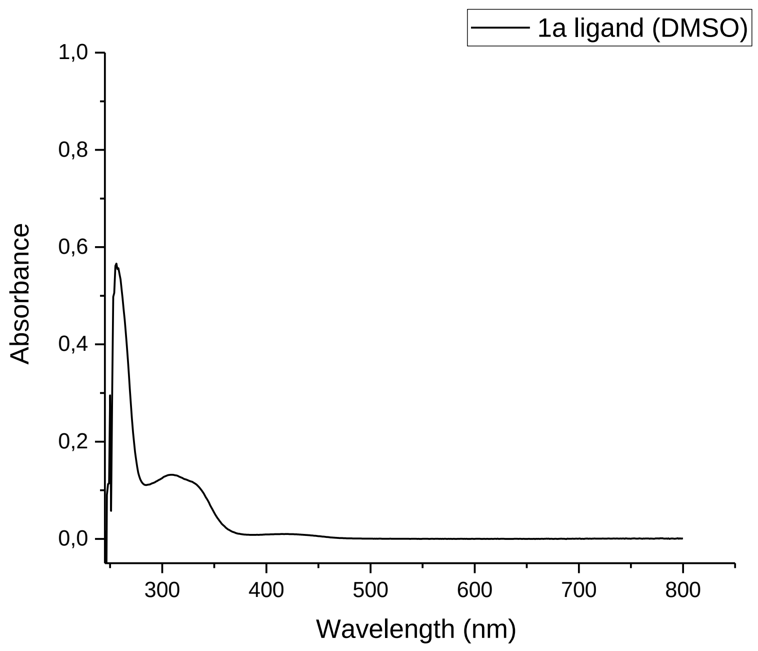

Figure S11. UV-Vis spectra of **1a** (1×10^{-7} M) in DMSO (250 – 800 nm).

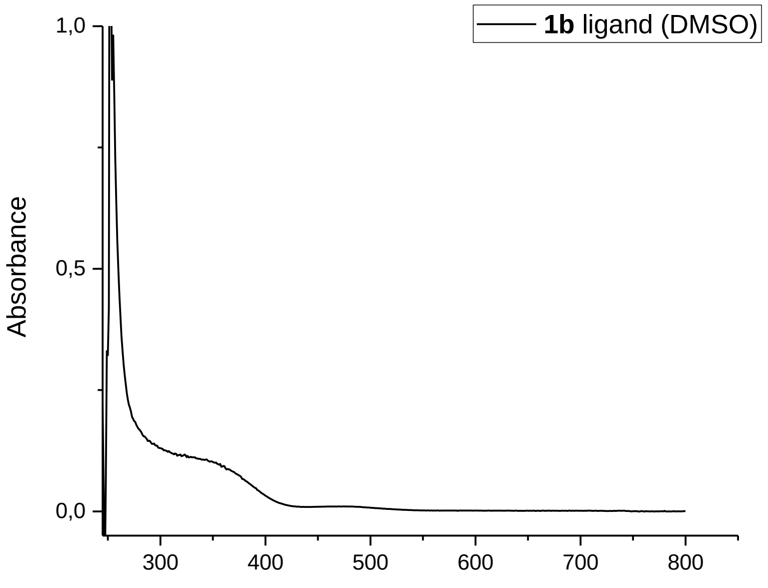

Figure S12. UV-Vis spectra of **1b** (1×10^{-7} M) in DMSO (250 – 800 nm).

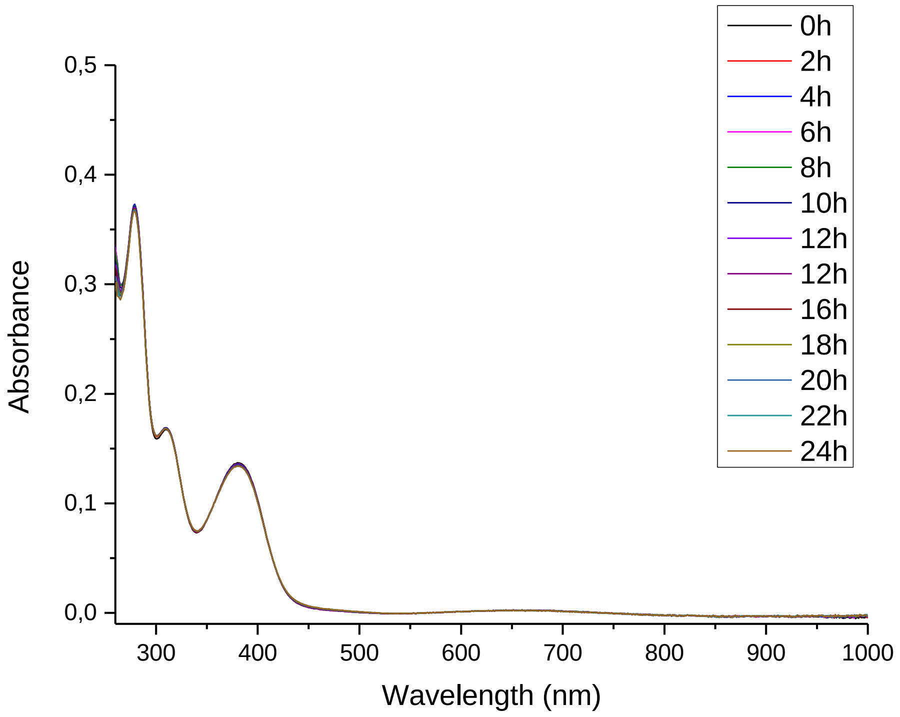

Figure S13. UV-Vis spectra of **2a** (1×10^{-7} M) in DMSO (250 – 1000 nm).

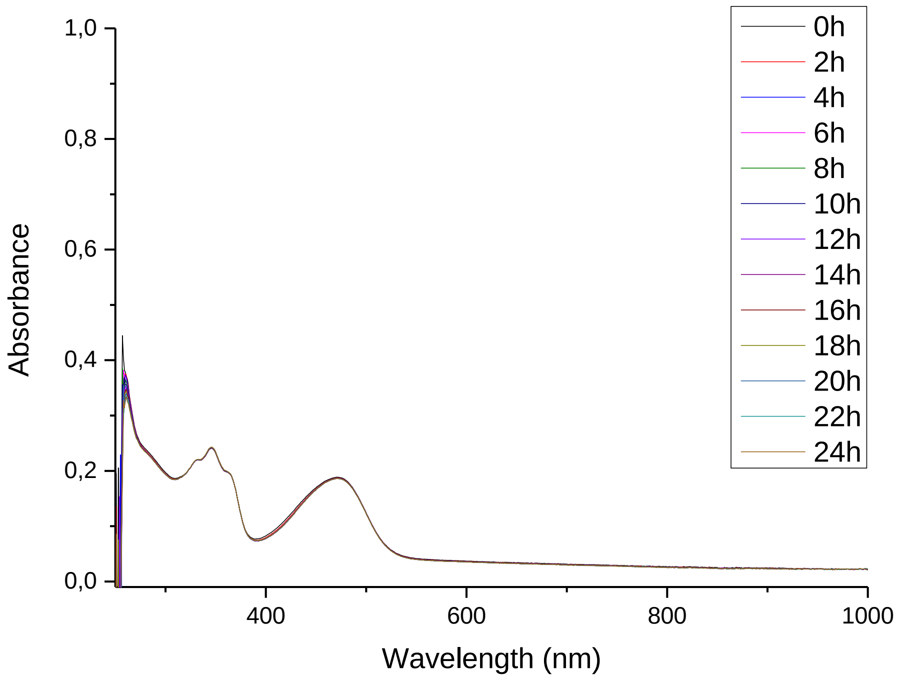

Figure S14. UV-Vis spectra of **2b** (1×10^{-7} M) in DMSO (250 – 1000 nm).

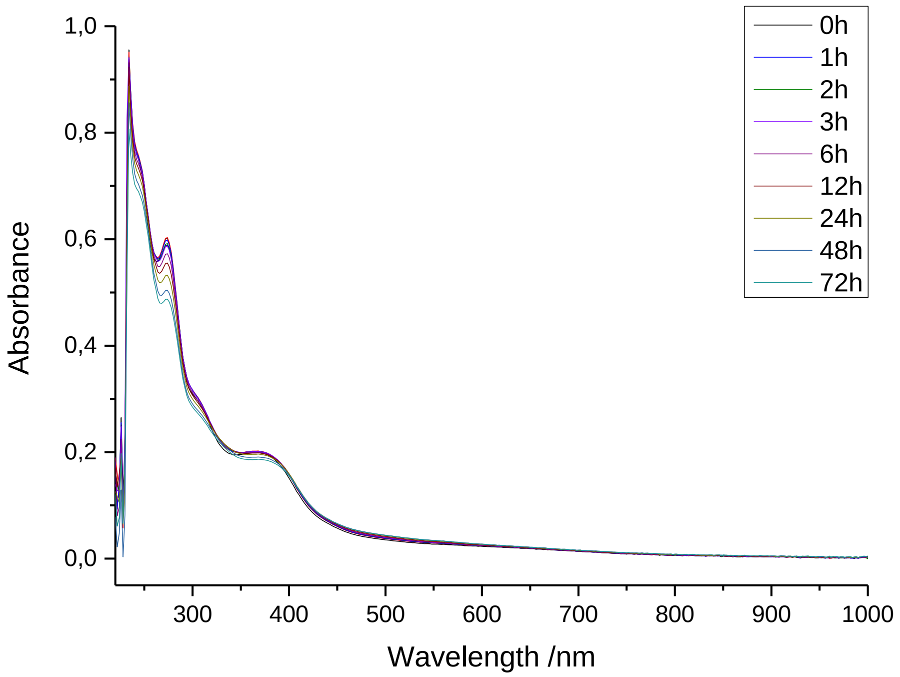

Figure S15. UV-Vis spectra of **2a** (1×10^{-7} M) in H_2O (250 – 1000 nm).

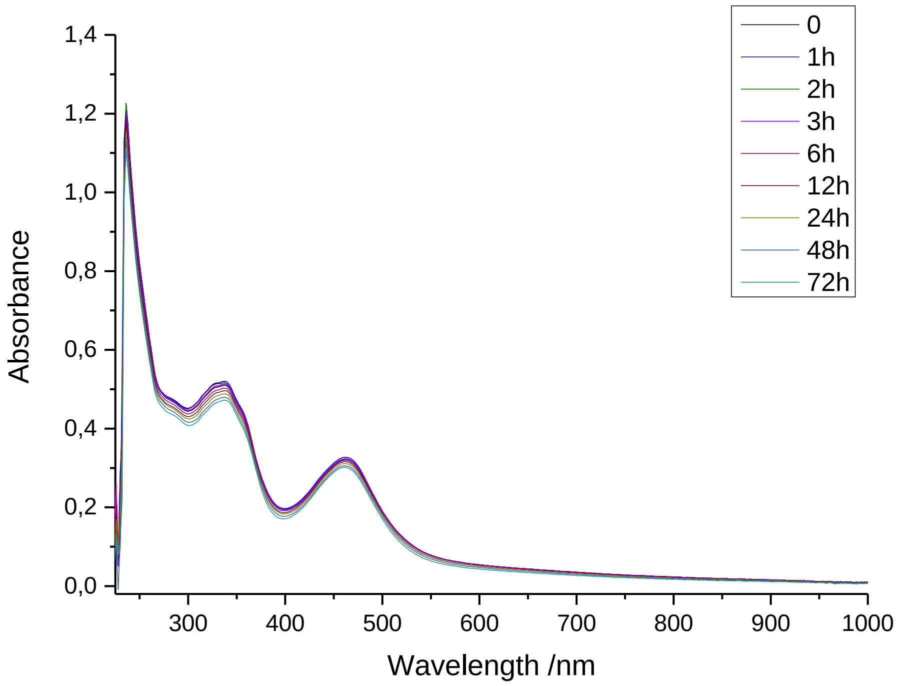

Figure S16. UV-Vis spectra of **2b** (1×10^{-7} M) in H_2O (250 – 1000 nm).

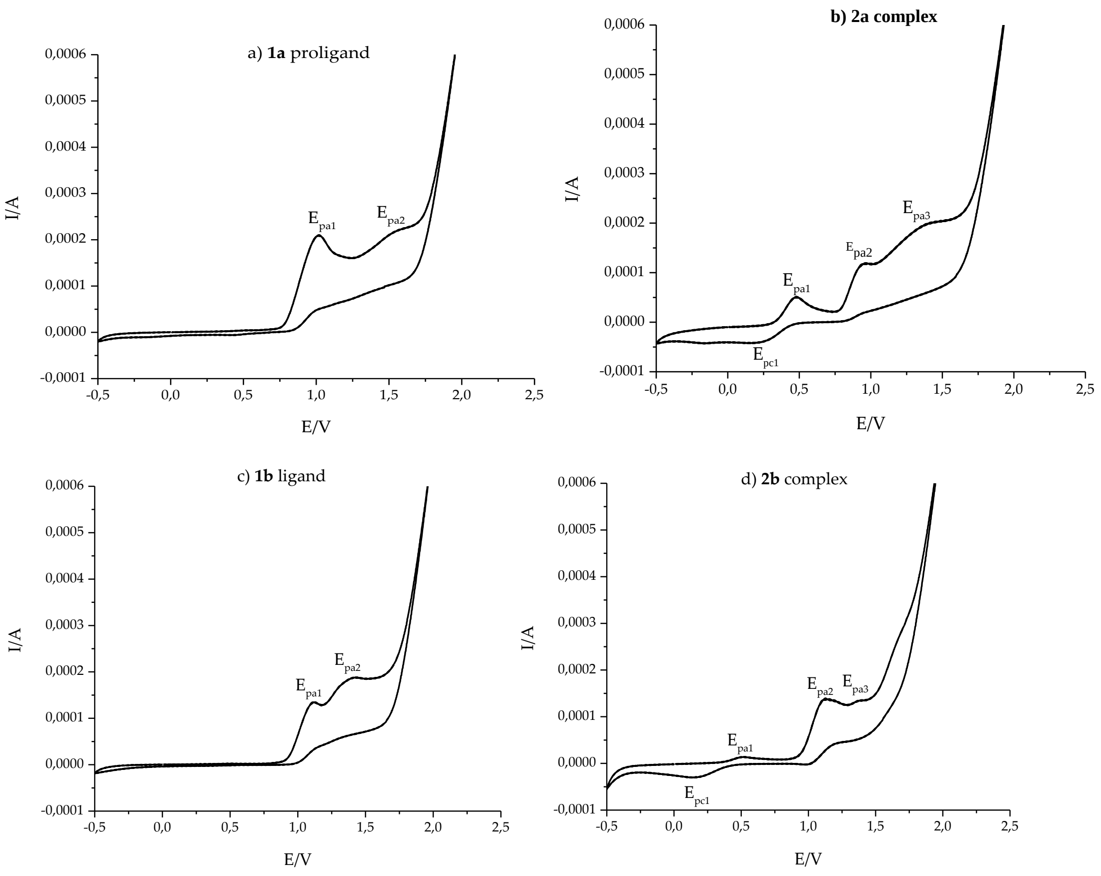

Figure S17. Cyclic voltammograms of ligands **1a**-**b** and Cu(II) complexes **2a**-**b**, in DMF solution at 100 mV/s scan rate, corresponding to the **1a** (a), **2a** (b), **1b** (c) and **2b** (d).

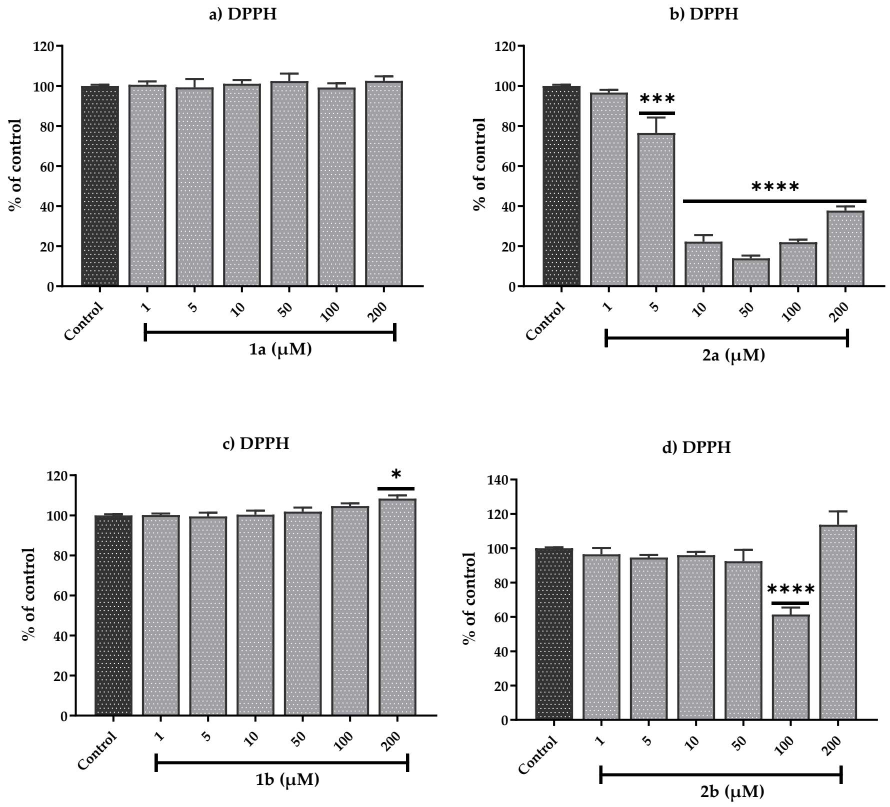

Figure S18. Effect of ligands and Cu(II) complexes represented by the 1a (a), 2a (b), 1b (c), 2b in different concentrations on DPPH radical-scavenger activity. Data are expressed as the mean ± SEM for three independent experiments, carried out on different days. The results are calculated as % of control of the absorbance at 517 nm. Asterisk denotes significance levels when compared to control group: (*) $P < 0.05$, (***) $P < 0.001$ and (****) $P < 0.0001$ (One-way ANOVA followed by the Newman-Keul's test).

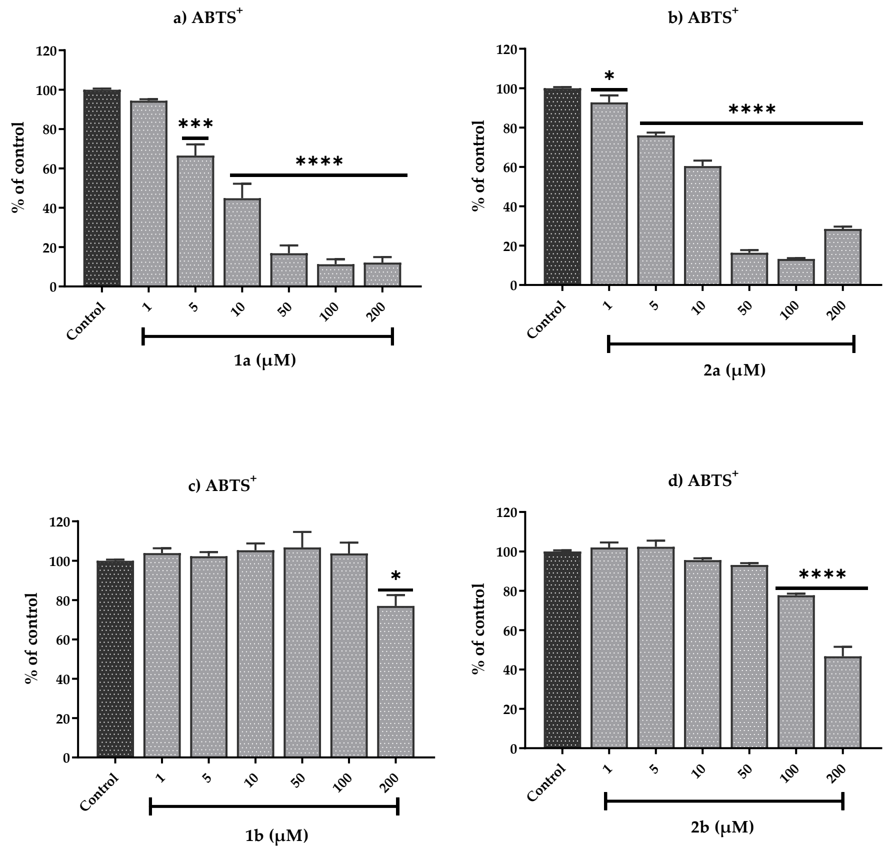

Figure S19. Effect of ligands and Cu(II) complexes represented by the **1a** (a), **2a** (b), **1b** (c), **2b** in different concentrations on ABTS^{+} radical-scavenger activity. Data are expressed as the mean ± SEM for three independent experiments, carried out on different days. The results are calculated as % of control of the absorbance at 730 nm. Asterisk denotes significance levels when compared to control group: (*) P < 0.05, (***) P < 0.001 and (****) P < 0.0001 (One-way ANOVA followed by the Newman-Keul's test).

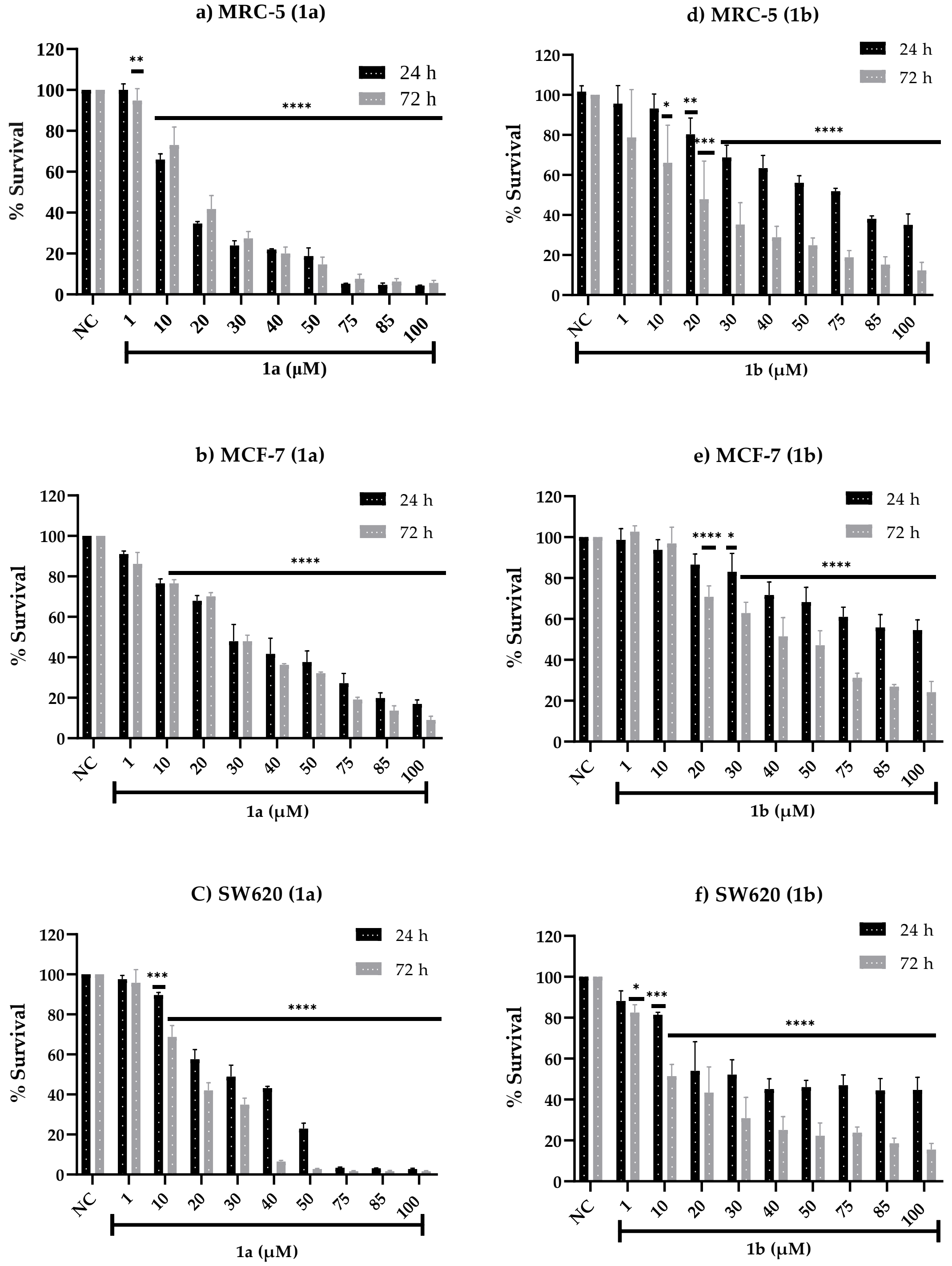

Figure S20. Comparison of the dose-response survival diagrams of MRC-5, MCF-7 and SW620 cell lines exposed to **1a** and **1b** free ligand (0 – 100 μM) for 24 h or 72 h. The obtained values represent an average of at least three independent experiments. NC represents the negative control. Graphs represent the average ± SD. Statistical analysis were performed using One-way ANOVA followed by

Dunnett's multiple comparison test and p < 0.05 was considered as significative. * p = 0.0189 (d), 0.0145 (e), 0.0224 (f); ** p = 0.0027 (a), 0.0015 (d); *** p = 0.0010 (c) and (f), 0.0003 (d); **** p = <0.0001.

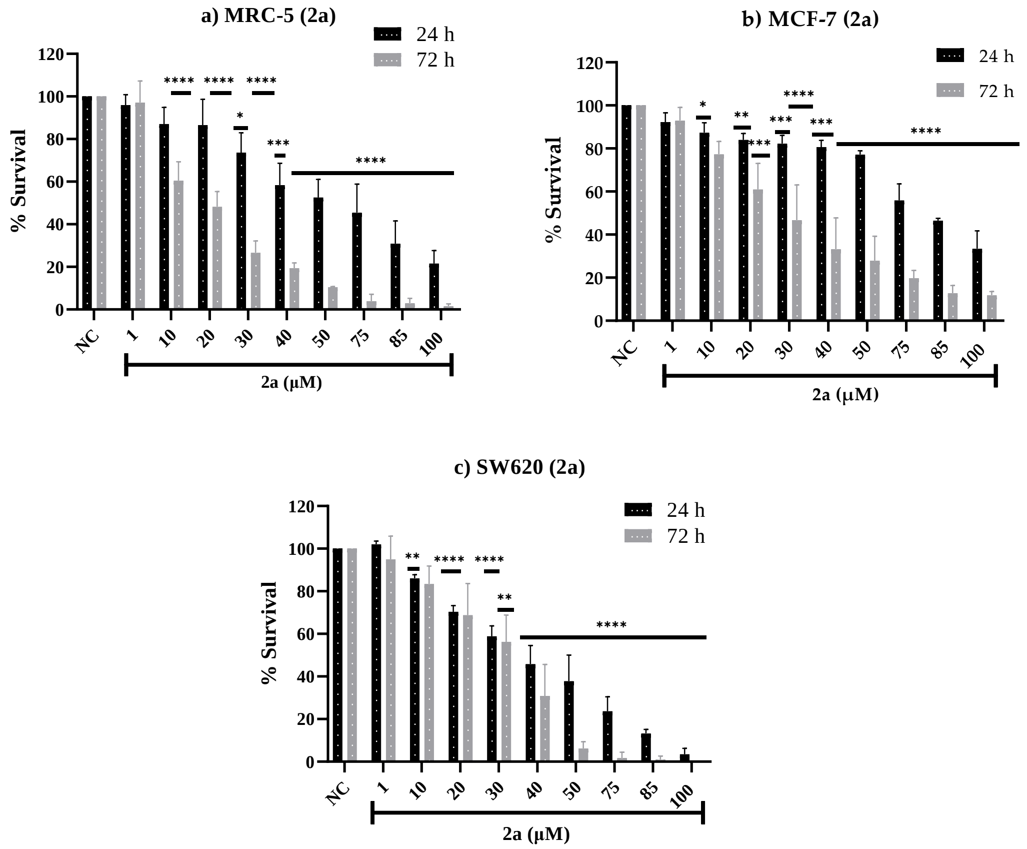

Figure S21. Comparison of the dose-response survival diagrams of MRC-5, MCF-7 and SW620 cell lines exposed to complex **2a** (0 – 100 μM) for 24 h and 72 h. The obtained values represent an average of at least three independent experiments. NC represents the negative control. Graphs represent the average ± SD. Statistical analysis were performed using One-way ANOVA followed by Dunnett's multiple comparison test and p < 0.05 was considered as significative. * p = 0.0141 (a); 0.0185 (24 h - b), 0.0445 (72 h –b), ** p = 0.0026 (b), 0.0036 (24 h - c), 0.0017 (72 h – c) *** p = 0.0002 (a); 0.0009 (24 h-b), 0.0004 (72 h –b), **** p = <0.0001.

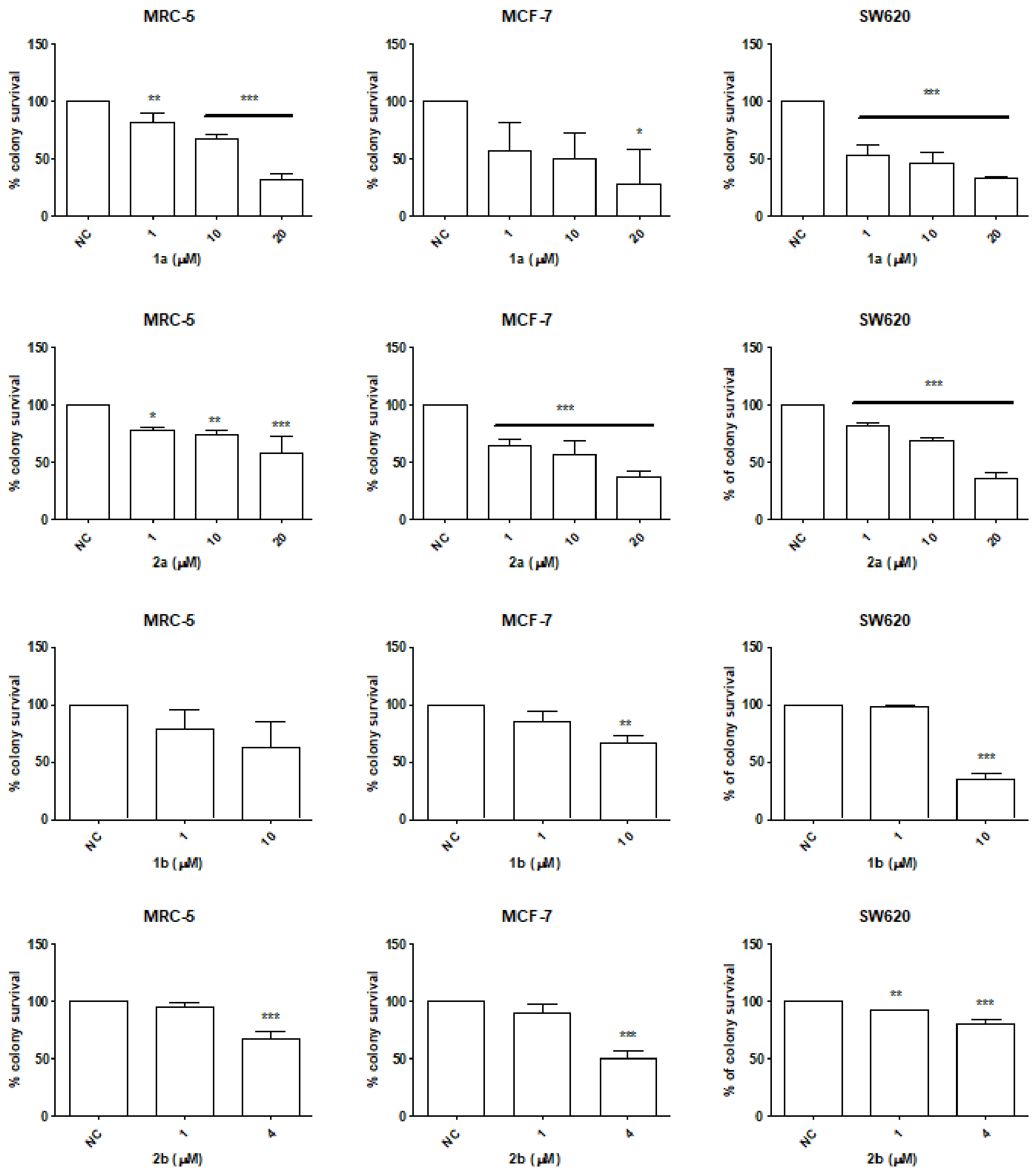

Figure S22. Colony Survival for **1a**, **1b**, **2a** and **2b** on MRC-5, MCF-7 and SW620. Data represent mean and SD of 3 independent experiments. One-way ANOVA and Dunnett1s posttest. (*)p<0.05; (**) p<0.01; (***) p<0.001.

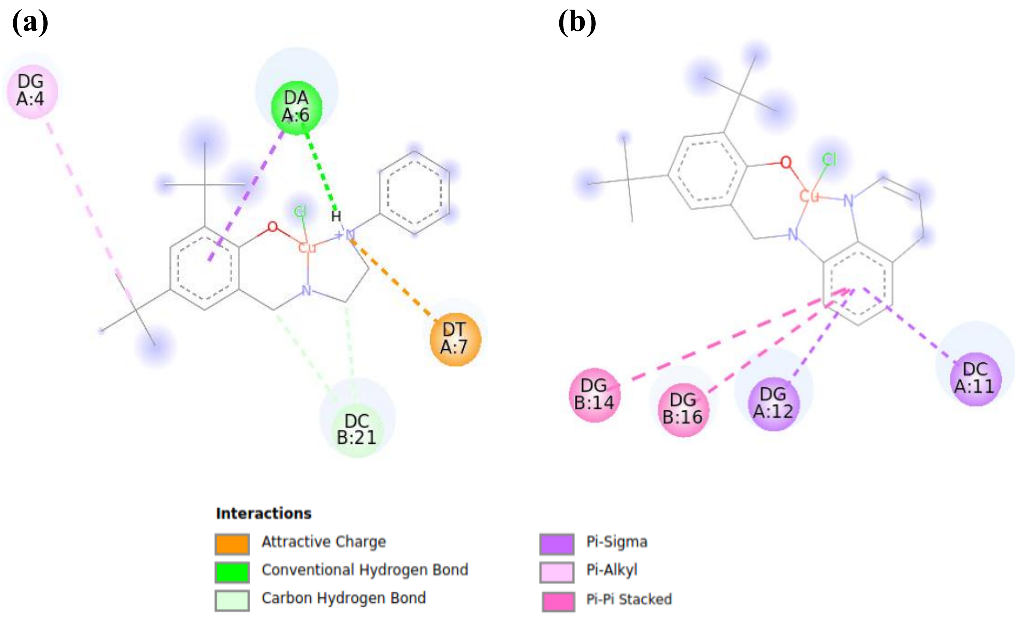

Figure S23. Two-dimensional interaction diagram of complexes **(a) 2a** and **(b) 2b** with DNA (PDB: 1BDNA).

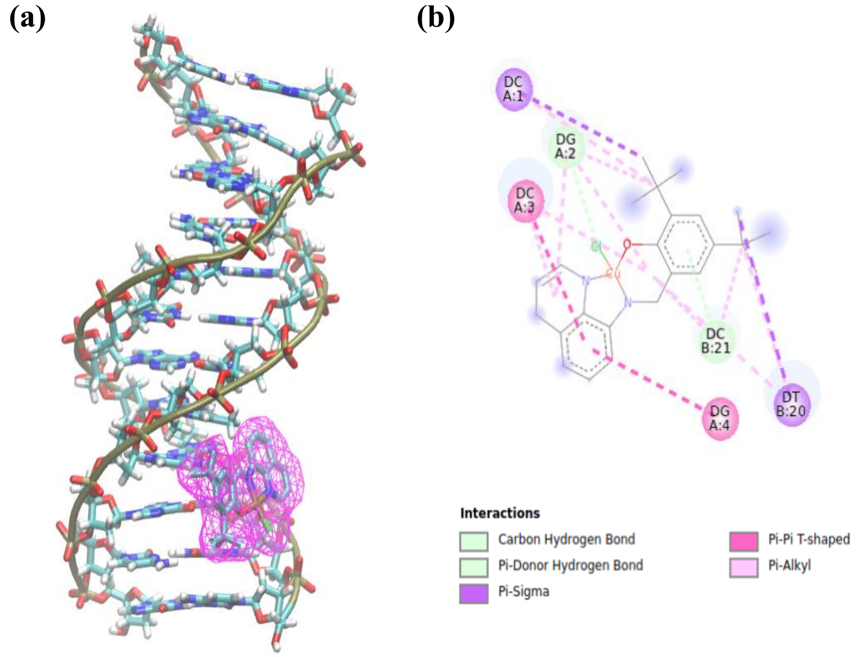

Figure S24. (a) An alternative docking pose for **2b** with an affinity value of –7.4 kcal mol^{-1}. **(b)** Two-dimensional interaction diagram of this mode with DNA.

Table S1. Crystallographic data and structure refinement parameters for **2a′**.

Complex	**2a′**
Empirical formula	$C_{92}H_{124}Cl_4Cu_4N_8O_4$
Formula weight (g mol^{-1})	1801.94
T (K)	111(2)
Crystal system	Triclinic
Space group	*P-1*
a (Ǻ)	11.4946(5)
b (Ǻ)	12.1360(5)
c (Ǻ)	16.7973(7)
	77.584(2)
	87.932(2)
	87.077(2)
V (Ǻ^3)	2284.66(17)
Z	1
Radiation type	Mo $K\alpha$
ϱ_{calcd} (g cm^{-3})	1.310
μ (mm^{-1})	1.088
F(000)	948
Crystal size (mm)	0.14 × 0.09 × 0.06
θ range (°)	2.327 to 30.686
Limiting indices (*h, k, l*)	$-16 \leq h \leq 16$
	$-17 \leq k \leq 17$
	$-24 \leq l \leq 24$
Reflections collected	77504
Reflections unique (R_{int})	14148 (0.0787)
Completeness to θ_{max} (%)	99.9
Data / restraints / param.	14148 / 0 / 517

Absorption correction	Multiscan
Min. and max. Transmission	0.7080 and 0.7461
R_1 [I > 2σ(I)]	0.0440
wR_2 [I > 2σ(I)]	0.0804
R_1 (all data)	0.0843
wR_2 (all data)	0.0902
S on F^2	1.011
Largest diff. peak and hole (e Å^{-3})	0.614 and −0.558

Table S2. Selected geometrical properties of compounds **2a**, **2a′** and **2b** at the B3LYP/def2-TZVPP/def2-SVP level of theory. Distances are in angstroms and angles in degrees.

	Calculate value		
**Parameter**	**2a**	**2a′**	**2b**
Cu-O	1.904	1.954	1.923
Cu-Cl	2.219	2.261	2.235
Cu-N1	1.937	1.962	2.000
Cu-X	2.109	2.162	2.062
Cu-Cu	-	3.417	-
Cu-Cl2	-	2.959	-
N1-Cu-Cl	163.4	174.4	171.2
X-Cu-O	169.6	175.2	169.4
O-Cu-Cl	98.8	92.9	94.2
X-Cu-Cl	88.1	91.9	94.6
N1-Cu-X	82.8	83.8	81.1
O-Cu-N1	92.2	91.4	90.9

Coordinates xyz : Coordinates from ORCA-job 2a

Cu	6.42927785072474	3.34919281219363	6.26155145588961
O	5.37803864012287	3.51217530414253	4.68240461520883
Cl	5.31985489493879	1.67369650160085	7.23939139736439
N	7.88888188813128	3.16793670642029	7.76649587423149
H	7.32664449521611	2.73934977925756	8.49607159581196
N	7.35152785036804	5.05935965447030	5.91696650202245
C	8.98139221043392	2.29197166969540	7.42812393099237
C	9.54058361259485	2.32135389754930	6.14460348989118
H	9.12776236535299	2.98743287630445	5.38517521094167
C	10.59766742193177	1.46567337941395	5.82753812579071
H	11.02550577660827	1.48844866669680	4.82258633710933
C	11.08962013726641	0.56840588879838	6.77981645377514
H	11.90951721948451	-0.10688642596945	6.52496782307364
C	10.51709010980218	0.52761278439325	8.05418215190476
H	10.88522165933636	-0.18092703297971	8.79989290873629
C	9.46845022240524	1.39019245480596	8.38015187793015
H	9.01536244460654	1.35955043540256	9.37435290893259
C	8.24584011723752	4.56476269618001	8.10947584906661
H	9.14332902228960	4.60652344349418	8.74841353062511
H	7.39526341827295	4.97848877567808	8.67157994532786
C	8.44949602069490	5.36608663237956	6.82792091957558
H	8.49745993546515	6.44375967532965	7.05980556067396
H	9.41179587229549	5.08790032233023	6.36497545266368
C	7.04384404226819	5.88469667159453	4.96534487391082
H	7.61182167685465	6.82797188856120	4.90353004210258
C	6.03210957020111	5.71521419994138	3.97569871287226
C	5.22705303838530	4.52566993589814	3.89099940974653

C	4.21050101489871	4.47406788331343	2.86482932854206
C	4.08860032813358	5.55978007009354	2.01369013679644
H	3.32349935204435	5.51500134994000	1.24049112139063
C	4.88582315587218	6.73753347782381	2.07003654211784
C	5.84283313438268	6.78777655292767	3.05845067163693
H	6.48791303104384	7.66096154041005	3.16925923610111
C	3.29982951727676	3.23957105428753	2.74300083546199
C	2.27433284818610	3.40144030994290	1.60723618211704
H	2.75733902441419	3.51562730648100	0.62346042616934
H	1.61133405700612	4.26622421679429	1.76930897598564
H	1.63983795620931	2.50306620090403	1.56063849932099
C	4.15090486245100	1.98766996175989	2.43236412870618
H	3.49625841877244	1.10822949716343	2.31730114153964
H	4.86655533325910	1.77980917853508	3.23769751550547
H	4.70741636565743	2.12228151204643	1.49016043659348
C	2.51504715032636	3.03148532765298	4.05859546891291
H	1.89913283050086	3.91746832328389	4.28479218274939
H	3.18617411499136	2.84265298506869	4.90560141670408
H	1.83851850787583	2.16701564881317	3.95692553750223
C	4.64347491251293	7.86416593313821	1.05622224105726
C	4.85409024441176	7.32120361719270	-0.37378964338876
H	4.68024329140262	8.11526588259184	-1.11882777217099
H	4.16422330880006	6.49447037962817	-0.60144519501813
H	5.88178873986967	6.94618746889469	-0.50301118456007
C	3.19656661955099	8.38370724239666	1.19802498774587
H	3.00244987303752	9.18990323477469	0.47125738327080
H	3.02106258687796	8.78233314126339	2.20976389163321

H	2.45738127284263	7.58825974921757	1.01814649036440
C	5.60299321481215	9.04476401594033	1.27061869571163
H	6.65551984096369	8.73988304782376	1.15916895720259
H	5.47924411919238	9.49561002178494	2.26793470467289
H	5.40399945913284	9.83037027452752	0.52477970345374

Coordinates from ORCA-job 2a′

Cu	10.43465165283363	7.72443878369060	7.87480873110712
O	10.46934505414110	7.64204537952991	5.92286193237510
N	10.30331607811301	7.96948435581608	10.01866883196358
H	11.30547997665298	8.05390016234102	10.17127335230819
Cl	11.74222640787630	5.89207070474505	8.09005927162183
C	9.79242933746797	6.85595643679011	10.74341724108252
N	9.20042094694973	9.24949505712336	7.83794981931180
C	8.41779399081223	6.68743258602720	10.96448401392852
H	7.70000304447882	7.41321693576422	10.58400872881750
C	7.95524258253351	5.57640967581781	11.67363316729596
H	6.88171332297797	5.45758592647420	11.83990904162293
C	8.85165986360905	4.62381651529686	12.16502988561267
H	8.48533191469536	3.75633653854877	12.71880007317175
C	10.22163803840571	4.79233753182995	11.93984954367101
H	10.93989849158612	4.06208270342929	12.31723727032841
C	10.69178918474088	5.89677642231889	11.23375672230019
H	11.75782452254933	6.01804910820786	11.05779529355457
C	9.66076920910438	9.29073340347329	10.21408282321649
H	10.46099469873845	10.03585621473906	10.14149301037518
H	9.20866702247339	9.34928403539009	11.21584349514592

C	8.62621342452340	9.57892624714562	9.12950849906117
H	7.72218870527822	8.96567896861147	9.27541297761618
H	8.31954268159097	10.63892526109815	9.17885420653413
C	9.15734288713332	10.07840327094894	6.85654177345167
H	8.66852838617756	11.05249727639400	7.02961201241230
C	9.65603132370584	9.87010521195645	5.52716369172593
C	10.33329922143805	9.55573489545241	2.86648583900816
H	10.57224303784928	9.43565078915100	1.81121712554317
C	10.47515409307211	8.45008265146111	3.69244554903803
C	10.20628841195109	8.61536364330337	5.10216742226675
C	9.89062424767634	10.83467395329260	3.29034320965460
C	9.52953688861286	10.95117937089058	4.61774960576319
H	9.13087356621972	11.88840125699040	5.00884497900562
C	10.85881362843363	7.06881883413091	3.13047692445287
C	12.15975549240363	6.54411427488402	3.77121039406956
H	12.45399312111749	5.59258457620546	3.29882594248416
H	12.01220377886979	6.35940374770301	4.84138366677536
H	12.98260616280003	7.26258211271377	3.63204839839710
C	9.70973356113215	6.07732466815860	3.42242189458599
H	8.77699534815833	6.41165583570555	2.94045075523132
H	9.53333098052036	5.98470152542046	4.50245872108915
H	9.96006478998405	5.07907708613653	3.02652760958949
C	11.07468148440051	7.11158673892870	1.60799601278315
H	10.17121213171690	7.43938024736764	1.07058646541654
H	11.32553339204883	6.10205389068588	1.24722753754760
H	11.90493579773042	7.77959948654336	1.32831133973206
C	9.79728723557653	11.98184778019748	2.27480731971581

C	8.83436438899564	11.58441606579163	1.13520969198527
H	8.74975316012200	12.39912404729559	0.39708245194823
H	7.82858407539300	11.37172474420615	1.53079308774696
H	9.18197844946543	10.68630965106551	0.60233711542097
C	11.19619851230450	12.26384788961314	1.68655891198396
H	11.62424040909364	11.37218317176925	1.20367052568696
H	11.89459350630463	12.58397306272383	2.47563283521790
H	11.14543330432712	13.06474507967311	0.93046437289592
C	9.27581302933289	13.27491447869224	2.91973605792016
H	9.23370905881590	14.07910065937242	2.16848731262676
H	9.93189400299473	13.61544727341165	3.73627919325433
H	8.26027720454690	13.14644928459320	3.32606469560370
Cu	13.77976776397461	7.98120986452543	8.52427744292796
O	13.74499091548372	8.06355675152856	10.47624690534671
N	13.91117621240375	7.73618140373525	6.38038136967057
H	12.90901854912799	7.65171665048832	6.22777907784875
Cl	12.47226949992673	9.81360478975128	8.30897643243940
C	14.42202067725026	8.84972155391136	5.65562823263368
N	15.01405873916921	6.45620843639089	8.56114448347256
C	15.79664909465609	9.01829646283023	5.43455511142808
H	16.51446868702221	8.29254265799702	5.81503525505140
C	16.25915572026303	10.12932969062000	4.72539276645857
H	17.33268016009839	10.24819137622248	4.55911223276898
C	15.36270135518074	11.08188136414985	4.23398333178205
H	15.72899415670359	11.94936735804204	3.68019925166406
C	13.99272974700452	10.91330777300864	4.45916516278507
H	13.27443807950637	11.64352555465819	4.08176333618692

C	13.52262407900030	9.80886076011430	5.16527483720171
H	12.45659340145221	9.68754858799568	5.34122952799783
C	14.55378761219148	6.41496220638805	6.18499742363074
H	13.75359533026540	5.66980239691471	6.25756530185474
H	15.00592217139553	6.35642204633768	5.18325058946525
C	15.58832849308134	6.12682747494576	7.26960008454309
H	16.49231551893693	6.74013796132542	7.12372895030625
H	15.89507057847287	5.06684899085571	7.22025523909458
C	15.05712143035757	5.62726961977935	9.54252798641226
H	15.54596443310263	4.65319212195922	9.36944524915846
C	14.55836005194985	5.83550586318653	10.87188720605234
C	13.88070185301932	6.14966787408342	13.53249206236074
H	13.64160278007102	6.26966943755615	14.58773514831830
C	13.73910484275968	7.25541986738192	12.70662104613791
C	14.00811148909471	7.09023776122826	11.29691908936957
C	14.32340571845401	4.87075254524154	13.10859265318957
C	14.68471081862833	4.75435748810573	11.78123409754416
H	15.08338605271634	3.81715155453353	11.39011289293862
C	13.35540729092908	8.63665249420471	13.26863523089579
C	12.05501515234178	9.16175855172504	12.62712241525665
H	11.76064189326627	10.11324464204137	13.09950775034625
H	12.20336697262172	9.34668120458224	11.55709456378720
H	11.23191529505544	8.44342636834124	12.76553271056880
C	14.50488885651513	9.62797479731585	12.97766837131574
H	15.43723753370595	9.29334639296367	13.46018766910503
H	14.68202787178454	9.72080251037297	11.89777493945452
H	14.25450109947601	10.62618645465298	13.37361940138731

C	13.13853530223132	8.59361523197141	14.79096547989562
H	14.04156755511071	8.26546111152236	15.32889092475046
H	12.88771798253762	9.60313777485758	15.15178692960825
H	12.30791926103346	7.92576820799152	15.06996818177773
C	14.41653019594876	3.72348050274844	14.12403684630939
C	15.37920606926862	4.12079937927183	15.26388166676807
H	15.46366430304058	3.30601476765755	16.00194148257876
H	16.38506955753893	4.33353870245292	14.86853565973144
H	15.03147258250653	5.01884731298736	15.79677463329066
C	13.01749121748567	3.44142160437223	14.71195521254297
H	12.58933371504668	4.33304729924867	15.19481349478688
H	12.31927764794868	3.12134774113858	13.92270045287139
H	13.06809502292465	2.64046697919532	15.46800003355532
C	14.93814564671746	2.43047758102100	13.47909378044248
H	14.98010308844023	1.62622103028823	14.23027530547268
H	14.28223259766531	2.09001580479760	12.66238637635128
H	15.95376205357689	2.55899181944360	13.07298233505245

Coordinates from ORCA-job 2b

C	3.98553773990649	6.66302105229965	1.23463602050195
C	5.38327466526945	6.80396263000328	1.22218671122103
C	3.19815729011710	7.78119750510912	1.23952938981237
H	3.55367588559393	5.67096324738992	1.24580298524992
N	5.96210211048391	7.98125637956060	1.20388141875383
H	6.06074494623846	5.95544934958883	1.22957676715870
C	3.79294542109280	9.05807288205004	1.22169472078716
H	2.11637710503687	7.70127529689300	1.25846559767834

C	5.20392293243370	9.10496026982697	1.19436918443910
C	3.06709429820248	10.26732029573773	1.23260099024928
C	5.89045564415590	10.34516238081256	1.15542949886932
C	3.74195752141210	11.45856498816616	1.22211004195239
H	1.98416122655670	10.23942752215003	1.26063213152278
C	5.14490289253038	11.50528754441293	1.18770647369919
H	3.18970355200002	12.39056565843129	1.24746750336903
H	5.63089640636212	12.47191723646744	1.20003307158971
N	7.27562300809065	10.23577207150709	1.10320426574922
C	8.02181525406793	11.27461916806792	0.84741291874034
Cu	7.96915488908284	8.38570556746848	1.23757267780680
O	9.76833168484542	9.03651884935333	1.38467638214298
Cl	8.58276831088914	6.25269634747506	1.21748178005582
C	9.42109379422556	11.32243549585911	0.80210392763049
H	7.52326343494743	12.21854589112523	0.62105214645677
C	10.24359577259055	10.19426220876209	1.12338640794372
C	11.66597691145564	10.41133416844828	1.14653065351856
C	12.14211888205153	11.63114617563511	0.73673448530945
C	9.99574541071862	12.55578674089626	0.40514297082043
C	11.34404467688048	12.72699565763078	0.32367288577803
H	13.21526050872356	11.76634342156093	0.72423185388723
H	9.31537852526167	13.35589605061243	0.13614352156063
C	11.99094461927697	13.97004166368386	-0.27201445190710
C	11.00533641950721	15.13273002031553	-0.36274594957050
C	12.46562483281251	13.61745727898008	-1.68823979765462
C	13.19221519747244	14.43074891385153	0.55493351561377
C	12.60757932120909	9.28677399820755	1.55969961675383

C	12.51193190179040	8.15136682847341	0.53237139127803
C	12.25860012325396	8.78100710926371	2.96570583272261
C	14.06161142624361	9.74924541947291	1.60693690457731
H	11.23926704118700	8.40509302945405	3.02238560737708
H	12.38666409444008	9.58284139581382	3.69849017009370
H	12.93616522041993	7.96759394784553	3.23765963248603
H	11.50770856333329	7.73265511630527	0.48717910300203
H	13.20745896506827	7.35050123075180	0.79711402202433
H	12.78565073592830	8.52335706045805	-0.45913783081651
H	10.16784134897535	14.90784818612187	-1.02720373455626
H	11.50977242781732	16.01280976755395	-0.76854119582348
H	10.60274105191760	15.39448383565105	0.61943898476211
H	11.62719385233265	13.28230862169517	-2.30339815755956
H	13.20479016389788	12.81305581713133	-1.66807522367061
H	12.92614251723201	14.48717110230930	-2.16620532638066
H	12.89292399728100	14.69188061976857	1.57316213016543
H	13.64095929456844	15.31533661998144	0.09524705119500
H	13.96923976709117	13.66704482586151	0.61201209567690
H	14.43513547019600	10.04828546879464	0.62388976115731
H	14.68253379553478	8.92157031431120	1.95661177943085
H	14.20421114998920	10.58141875464114	2.30159468536842

Coordinates from ORCA-job 1a

C	-5.76873542475317	-3.01600485810156	-0.55615197992032
C	-5.07865064961583	-2.03396438797473	-1.27826845096544
C	-5.13166498658434	-4.19068782856504	-0.16760537183593
C	-3.77794610208207	-4.41939511750174	-0.49302011733659

C	-3.74069935664138	-2.25711932957272	-1.60731011753029
C	-3.09060377809309	-3.43558684976981	-1.22915722756585
N	-3.15884557788085	-5.58365363660585	-0.05406140685152
H	-5.67680607980246	-4.94738471158812	0.40474327250354
H	-3.18570375542786	-1.50555692527880	-2.17537697262406
H	-2.04712830644209	-3.58394760538480	-1.50840553429700
H	-6.81813963211316	-2.86355985388598	-0.28970861936217
H	-5.57981214855101	-1.11193804867564	-1.58068225665684
C	-1.86967972361405	-6.00457053044944	-0.55670630783278
C	-1.43361939373650	-7.30113704045261	0.12470717799750
H	-1.11816728807566	-5.22926480749641	-0.33706535651882
H	-1.86970447074520	-6.14671841527182	-1.65697082612437
N	-0.11050278617286	-7.67952078452965	-0.31428356469591
H	-2.17284112157728	-8.10487402170485	-0.07506738361360
H	-1.41354795758161	-7.13181871305537	1.21544919445920
H	-3.79585666651217	-6.33274105339922	0.16851962974165
C	0.09381827241168	-8.83296289019925	-0.83000248889891
C	1.39528927042546	-9.27394348048732	-1.30552706772342
H	-0.73751497066890	-9.55597777637455	-0.93709988693530
C	1.52156674052089	-10.57148262000562	-1.84137901898776
C	2.52441027438987	-8.41592880224982	-1.24156332326088
C	3.78119540925302	-8.86191797726668	-1.73040968130110
C	3.83583404904978	-10.15791703695106	-2.24615059682170
C	2.73761406504418	-11.04235383484329	-2.31640960324860
C	5.02249707061735	-7.95138204546916	-1.69158635241013
C	4.75937188652548	-6.67429363421922	-2.52137945227804
H	5.65328429644768	-6.02896755086223	-2.51337152860594

H	3.91555273538117	-6.09851942272574	-2.12141669124465
H	4.53755355670429	-6.93335349012346	-3.56931745656186
C	6.25948792840200	-8.64650514175486	-2.28599843813859
H	6.11300383747849	-8.92028244784690	-3.34272221966337
H	6.53285953542827	-9.55581572455806	-1.72776952178121
H	7.11905767623236	-7.96027419971329	-2.23841440995186
C	5.35262121858284	-7.57777594769979	-0.22909131598848
H	4.52803158105994	-7.03148804267364	0.24568677868524
H	6.25287474802874	-6.94213533023870	-0.19661208962132
H	5.55578190875217	-8.48388449427531	0.36428800295902
C	2.93123813791773	-12.45034654889365	-2.89886246041705
C	1.62375754498788	-13.25760461323949	-2.89491680013267
H	1.23109320942800	-13.39235784211609	-1.87480591996247
H	1.80122159009025	-14.25897221505633	-3.31734516038920
H	0.84334525130850	-12.77392181628267	-3.50317803374388
C	3.42944702757836	-12.34043661518122	-4.35583715559968
H	2.69713738071484	-11.80482379758532	-4.98051816461021
H	3.58388743882321	-13.34272325220607	-4.78824405358163
H	4.38502382333903	-11.79817326086303	-4.42000970796389
C	3.97440084009109	-13.21432008375234	-2.05539918246493
H	4.94803994937678	-12.70098190969012	-2.04724666379266
H	4.13347024887755	-14.22692125784523	-2.46143431473032
H	3.63778783105433	-13.31156384556157	-1.01103518749622
O	2.40150828593327	-7.18103049737498	-0.72104813112551
H	1.44515362511627	-7.06800085479972	-0.43748313676966
H	0.62752367168914	-11.19603966842292	-1.86864514313905
H	4.79293825960961	-10.51508551132678	-2.62125220327241

Coordinates from ORCA-job 1b

C	-1.51126892104837	-1.71536217644799	0.06007084592919
C	-0.96607743666408	-0.44746220420035	0.06482917412502
C	0.43159471747571	-0.26435255144964	0.01762858540136
C	-0.66256180768569	-2.85136756034290	0.01119464166954
C	0.76318774577102	-2.68563827469513	-0.00694766703349
C	1.30493770513417	-1.34429132643233	0.00401133267488
C	-1.16977730623653	-4.17840311963205	-0.01399364359395
N	1.59776940028339	-3.76525939502895	-0.05998999118927
C	-0.30131970259754	-5.24377126633774	-0.06312552440155
C	1.08882914601556	-4.97694060254436	-0.09178083438492
N	2.67725744632446	-1.14263427328488	-0.12503970434531
C	3.46183689189777	-1.83401924330526	0.60143329411758
C	4.89983046962942	-1.98937225346698	0.35330911860178
C	5.77059914971497	-0.91180568379629	0.14500368742550
C	7.13366115949083	-1.13318286092396	-0.04346419755304
C	7.57953659321901	-2.46925486735998	-0.02761446492605
C	5.36774398071226	-3.31714152612199	0.31056212546311
C	6.74627179903172	-3.58189729065572	0.13515111299511
C	7.29612126332579	-5.01956950073973	0.10394136447126
C	6.68962305751876	-5.78579751984904	-1.09317764001037
H	5.59604330497646	-5.84309799475630	-1.02413861406462
H	7.08957962569811	-6.81292067188988	-1.12487272267753
H	6.95144655441288	-5.28988803579371	-2.04161660804983
C	8.82617671214484	-5.04483066191218	-0.05522218334018
H	9.15098256840552	-4.58343697088078	-1.00072934850980

H	9.17314440702594	-6.08981666048611	-0.06139365446631
H	9.33687807622345	-4.53026796509250	0.77383500175058
C	6.95097639717442	-5.74003704705775	1.42621221543906
H	5.86669169386601	-5.79689354543463	1.58553489164470
H	7.39954658898759	-5.21133027864559	2.28274153437669
H	7.35283059184869	-6.76665448713425	1.41268986056788
C	8.14729409907312	0.00180225841088	-0.25875077572455
C	7.47479583003611	1.38317834548802	-0.23371323365363
H	6.71623598075229	1.48201918171741	-1.02569465400386
H	8.22914741934066	2.16951818492991	-0.39430017995531
H	6.98766580184048	1.58172350380266	0.73377269925890
C	8.83297494067217	-0.17906460845630	-1.62993818877689
H	8.09096529772950	-0.15461139082173	-2.44357433121568
H	9.36947692572636	-1.13807141150063	-1.69475104482545
H	9.56490531313375	0.62654805546111	-1.80677183072164
C	9.21128782809747	-0.04084911940203	0.85841475246003
H	8.74403433415822	0.08494130625098	1.84801616362205
H	9.95044042689659	0.76595481991225	0.72170851762987
H	9.75702650570513	-0.99663462198422	0.86492111371703
H	5.34627095455856	0.09128912548787	0.15808254289298
H	8.64406440983560	-2.64686739687003	-0.16552880602504
H	3.06468234368940	-2.44194532186172	1.43179968156867
H	-2.25215719933334	-4.33260413825986	0.00296709772511
H	-0.65917525824524	-6.27482591856575	-0.08621827554607
H	1.80440408634190	-5.80504236850070	-0.14384177600049
H	0.85793329791735	0.74037002120719	-0.00473875873035
H	-2.59350407473455	-1.86327035520057	0.08987620861022

H	-1.61811499487258	0.42884797873453	0.09723801940000
O	4.47738581684845	-4.35182433827602	0.41150325658731
H	3.56110804275585	-4.05166397600421	0.21448981359975
